# Traumatic experiences in childhood and psychopathy: a study on a sample of violent offenders from Italy

**DOI:** 10.3402/ejpt.v4i0.21471

**Published:** 2013-12-20

**Authors:** Giuseppe Craparo, Adriano Schimmenti, Vincenzo Caretti

**Affiliations:** 1 Faculty of Human and Social Sciences, University of Enna “Kore”, Enna, Italy; 2 Department of Psychology, University of Palermo, Palermo, Italy

**Keywords:** Psychopathy, child abuse and neglect, violent offenders, high-risk groups

## Abstract

**Background:**

The link between early traumatic experiences of abuse/neglect and criminal behaviour has been widely demonstrated. Less is known, however, about the relationship between these experiences and the development of psychopathic personality.

**Objective:**

This study investigated childhood relational trauma in a group of violent offenders from Italy. We hypothesised a higher level of early relational trauma associated with higher scores on psychopathy.

**Method:**

Twenty-two offenders convicted for violent crimes aged 22–60 (M=38, SD=11) participated in this study. Participants were selected by the Italian justice system for an experimental research programme aiming at the evaluation of psychopathic personality traits among violent offenders. Within the group, 14 participants (64%) had committed murder, 4 (18%) had committed rape, and 4 (18%) were convicted child sex offenders. The Traumatic Experience Checklist was used to assess childhood relational trauma; the Hare Psychopathy Checklist—Revised (PCL-R) was used to assess psychopathy.

**Results:**

There was a high prevalence of childhood experiences of neglect and abuse among the offenders. Higher levels of childhood relational trauma were found among participants who obtained high scores on the PCL-R. There was also a significant negative association between age of first relational trauma and psychopathy scores.

**Conclusions:**

Findings of this study suggest that an early exposure to relational trauma in childhood can play a relevant role in the development of more severe psychopathic traits.

Psychopathy is a complex personality disorder, characterising individuals with emotional deficits who lack a regard for social norms, empathy, and remorse (Hare, 1991, 1993). In the early years, Cleckley (1976) described the inability to participate in, or understand, the emotional aspects of humanity as one of the fundamental factors in psychopathy:

Let us say that, despite his otherwise perfect functioning, the major emotional accompaniments are absent or so attenuated as to count for little …. If we grant the existence of a far reaching and persistent blocking, absence, deficit, or dissociation of this sort, we have all that is needed, at the present level of our inquiry, to account for the psychopath. (p. 371).

Robert Hare shed light on the affective and interpersonal-related issues linked to psychopathy. His approach—operationalised in the golden standard Hare Psychopathy Checklist—Revised (PCL-R; Hare, 2003)—contributed to the understanding of the origins and maintenance of the disorder leading to the important distinction between psychopathy and antisocial personality disorder (Rutherford, Cacciola, & Alterman, 1999). The PCL-R enabled the distinction of psychopathy from antisocial personality disorder through specific traits, such as emotional detachment, grandiose sense of self-worth, manipulativeness, lack of empathy, superficial charm, shallow affect, parasitic lifestyle, irresponsibility, impulsivity, and social deviance (Cooke & Michie, 2001).

The characteristics of psychopathy identified by the PCL-R contributed to the understanding of high recidivism rates in psychopathic samples. In their study of 93 released prisoners, Serin and colleagues (1990) demonstrated that the severity of psychopathy was correlated with recidivism. Hart and colleagues (1988) showed that out of 231 individuals on parole, only 38% with a diagnosis of psychopathy and only 54% with a mild level of psychopathy were not reconvicted a year after. This finding was corroborated by Hare and colleagues (2000) who highlighted—in a sample of 728 offenders—that 81.8% of psychopaths were found guilty for another crime within 2 years after release.

Karpman (1941), Lykken (1995), and Porter (1996) put forward the idea of psychopathy as a result of dysfunctional interpersonal exchanges and adverse environmental factors, including child abuse and neglect. This is mirrored in more recent theoretical and clinical research on attachment theory (Bowlby, 1969, 1973, 1980) suggesting a relationship between child abuse/neglect and psychopathy (Caretti & Craparo, 2010). However, research in this field is still scant, and there are few studies demonstrating that psychopathy may be linked to a disruptive developmental history, including experiences of early trauma (Lang, Klinteberg, & Alm, 2002; Marshall & Cooke, 1999).

Thus, the causal role of early traumatic exposure in predisposing an individual to criminal behaviour has been well demonstrated (Ardino, 2011Ardino, 2012; Caretti, Ciulla & Schimmenti, 2011; Craparo, Faraci, Rotondo, & Gori, in press; Maxfield & Widom, 1996; Widom, 1989), whereas the link between early adverse childhood experiences and psychopathy remains controversial (DiLalla & Gottesman, 1991).

Some literature suggests a specific link between abuse and psychopathic features. More specifically, some authors have hypothesised that abuse results in a diminished capacity to respond with empathy. For example, in discussing the possible mechanisms by which psychopathy mediated the abuse–violence association in their sample, Weiler and Widom (1996) suggested that as a result of early abuse, “a child might become ‘desensitised’ to future painful or anxiety provoking experiences” and that this desensitisation might make “him or her less emotionally and physiologically responsive to the needs of others, to be callous and lack empathy, and to lack remorse or guilt” (p. 264); similarly, Porter (1996) indicated that the “capacity for empathetic responding … is ‘turned off’ with repeated disillusionment of the child through physical or sexual abuse or other mistreatment … the child's emotion being dissociated from or unconnected with cognition and behaviour over time” (p. 183). Porter's theorising implies that traumatic events can trigger a dissociation of affective capacities.

The aim of this study was to explore the relationship between an early traumatic exposure and a later development of psychopathy. In detail, the study aims to investigate the prevalence of early traumatic experiences in a group of violent convicted offenders and the association between early traumatic exposure and severity of psychopathy as measured by the PCL-R. It was hypothesised that individuals with an experience of early trauma would have scored higher in psychopathy levels as measured by the PCL-R.

## Method

### Participants

Participants of this study included 22 convicted male offenders aged between 22 and 60 [mean age (M): 38.05; SD=10.76], all diagnosed with antisocial personality disorder. Among them, 14 had committed murder (64%); 4 had committed rape (18%); and 4 (18%) were convicted child sex offenders. They were selected from a wider sample recruited for a comprehensive research programme commissioned by the Italian Ministry of Justice aimed at the evaluation of psychopathic personality traits within the criminal justice system.

### Measures

#### Traumatic Experience Checklist

Traumatic experiences were assessed with the Traumatic Experience Checklist (TEC; Nijenhuis, Van der Hart, & Kruger, 2002), a self-reported measure addressing 29 types of potentially traumatic events. TEC is a reliable and valid self-reported measure that can be used in both clinical practice and research. Different scores can be calculated including a cumulative score, and scores for emotional neglect, emotional abuse, physical abuse, and sexual abuse. The TEC has demonstrated good convergent validity, being associated with alleged reports and official records of traumatic experiences. In this sample, good reliability has been obtained for groups of items related to different types of abuse/neglect with a KR-20 reliability index of 0.65 or even more for groups of only three items.

#### Hare Psychopathy Checklist Revised

Psychopathy traits were assessed with the PCL-R (Hare, 2003), a 20-item measure scored on the basis of an interview and on file information. Each item is scored as 0 (not present), 1 (possibly present), or 2 (definitely present), resulting in total PCL-R scores that range from 0 to 40. The PCL-R has demonstrated good internal consistency, test–retest, and inter-rater reliability across diverse populations (e.g. Alterman, Cacciola, & Rutherford, 1993; Hare et al., 1990; Vitale, Smith, Brinkley, & Newman, 2002). The validation of the Italian version of the PCL-R (Caretti et al. 2011) showed similar psychometric properties, with a good internal consistency (all scales had a Cronbach's *α* >0.85), good inter-rater reliability [all intraclass coefficent correlation (ICC) above 0.90] and good convergent validity with other measures of personality and psychopathy. In this sample, Cronbach's *α* was 0.93 for the full scale, with *α* of factors and facets ranging from 0.85 to 0.93. Two independent raters scored the PCL-R, and the ICC for the full scale resulted as 0.94.

### Procedure

All participants were introduced to the aim of the study and were briefed to ensure researchers that they had understood all the steps involved in this research. It was explained to them that data were going to be recorded according to a strict procedure to guarantee the confidentiality of information. Data were kept anonymous by the substitution of the participants’ names with a code. Researchers briefed participants that they had the right to withdraw from the study at any time and to request to delete their responses from the database. All participants had to sign an informed consent prior to undertaking the study.

The study was ethically cleared by the Italian Ministry of Justice and by an ethics committee within the prison. The measures were administered individually in the presence of one of the researchers in a quiet room where prisoners usually receive family visits. For safety reasons, a police officer was available close to the room.

### Statistical analyses

Descriptive statistics were computed for all variables investigated in the study. Independent-samples *t*-test and Pearson's Chi-square test were used to compare participants with high and low scores on the PCL-R. The statistical package SPSS 19.0 for Windows was used for all the analyses (SPSS, Chicago, IL, USA).

### Results

We first checked for asymmetry and kurtosis in PCL-R scores; PCL-R scores were normally distributed in the sample. The PCL-R mean score was 19.49 (SD=7.71); median was 20.

Research with PCL-R usually showed three distinct groups, with participants scoring between 0 and 19 considered to have low psychopathy, participants with scores ranging between 20 and 29 considered as having medium psychopathy, and participants obtaining scores of 30 or more considered as severe psychopaths; however, in our sample only one participant obtained a score of 30 on the PCL-R. Thus, due to the reduced sample size, we lowered the PCL-R cut-off score to 25, after plotting the PCL-R scores and splitting the distribution of PCL-R scores at the nearest point to the 50th percentile of PCL-R scores between 20 and 30: we considered scores of 25 or more to the PCL-R as indicating high-risk for psychopathy (HRP). Eight participants (36.4%) scored 25 or more. This high-risk group was made up of five convicted murderers (35.7%, within the murderers sub-group), one rapist (25%), and two paedophiles (50%).

All 22 participants reported having had at least one traumatic experience in their own life. Among them, 17 participants (77.3%) reported early traumatic experiences (emotional neglect and/or physical, sexual, emotional abuse). In more detail, 4 (18.2%) reported sexual abuse, 11 (50%) reported physical abuse, 9 (40.9%) reported emotional abuse, and 15 (68.2%) reported emotional neglect (see Table 1). Twelve of the participants (55.5%) experienced at least two forms of abuse.

**Table 1:** Descriptive statistics

	M (SD)	Range
PCL-R		
Total score	19.49 (7.71)	2–30
Factor 1 (interpersonal/affective)	9.05 (4.23)	0–15
Factor 2 (social deviance)	8.76 (4.91)	1–18
Interpersonal	3.73 (2.37)	0–7
Affective	5.36 (2.61)	0–8
Lifestyle	4.91 (2.56)	1–8
Antisocial	3.85 (3.06)	0.10
TEC		
Total score	7.77 (4.99)	1–17
Emotional neglect	1.27 (1.03)	0–3
Emotional abuse	0.55 (0.74)	0–2
Physical abuse	0.95 (0.90)	0–3
Sexual abuse	0.36 (0.79)	0–3
Childhood trauma impact	3.24 (1.21)	1–4.80
Age of earliest trauma	9.41 (8.43)	2–40

**Figure 1:**
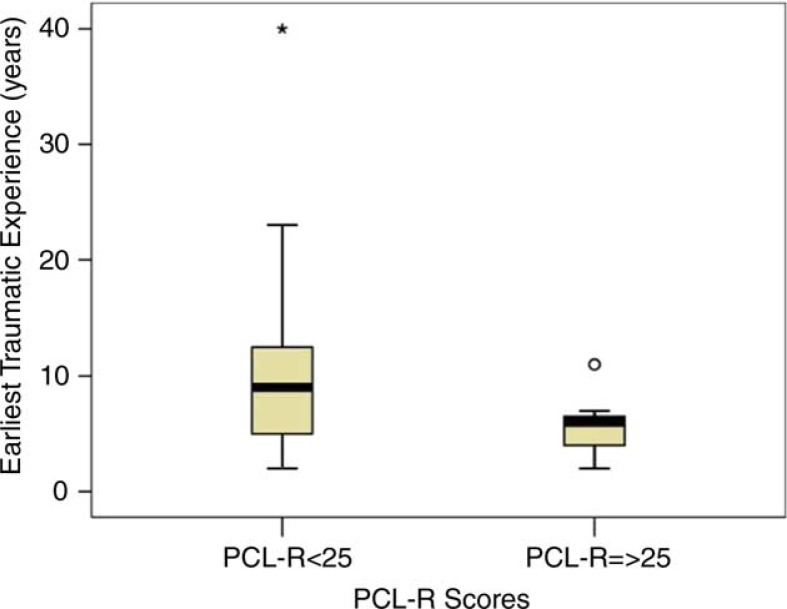
Boxplot representing the age in which participants experienced their first traumatic experience.

**Figure 2:**
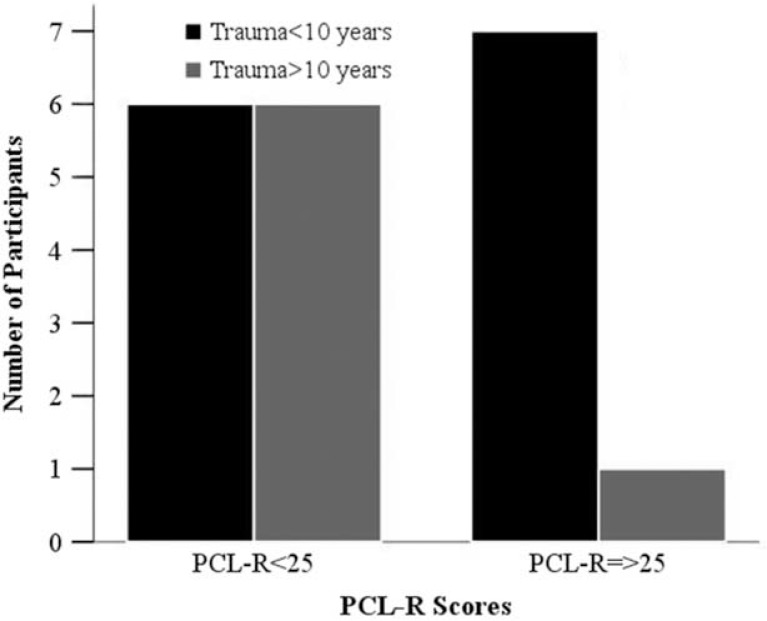
Childhood trauma among participants.

There was some evidence that the risk for psychopathy increases when trauma occurs early in life. Results showed that people in the HRP subgroup tended to have experienced a relational traumatic event earlier on in life compared to the rest of participants (mean age: 5.6, SD=2.85 vs. 11.5, SD=8.86; *t*(20)=2.18, *p*=0.05; Fig. 1). Data also showed that seven out of eight participants (87.5%) in the HRP group experienced a relational trauma before the age of 10, which is the age in which children in Italy terminate their primary education. The Chi-square test showed that people in the HRP group were more likely to experience traumatic events before this age, in respect with other participants [χ^2^(1)=4.20, *p*=0.040; Fig. 2]. These were mostly related to abuse and neglect in family environments (6 out of 7).

Although such findings do not imply a direct cause-effect between experiences of early relational trauma and psychopathy, results suggest a relationship between relational trauma in childhood and the development of psychopathic traits; our study indicates this is an important area (the relationship between early relational trauma and psychopathy) to be further explored in future studies.

### Discussion

Psychopathy represents a complex construct and is the subject of a major clinical debate. Theory and empirical research maintain that psychopathy may be linked to a history of trauma.

Among studies referring to the heterogeneity of psychopathy, Porter (1996)—following Karpman's (1941) distinction—has proposed that primary psychopathy mainly reflects a congenital affective deficit whereas secondary psychopathy reflects a detachment of emotions resulting from negative childhood experiences and acquired affective disturbances. Also, Blackburn (2009; Blackburn, Logan, Donnelly, & Renwick, 2008) has defined a typology in which two subtypes of psychopaths (secondary psychopaths and inhibited psychopaths) were more associated with high anxiety and withdrawal. These subtypes have been shown to be associated with a history of trauma. Therefore, analysing the relationship between traumatic stress symptoms and the various facets of psychopathy is of particular relevance in terms of clinical assessment and treatment.

The study explored this link by investigating the role of childhood experiences of abuse and neglect in individuals who presented criminal behaviours. Unlike a study published by Pham (2012), in which psychopaths reported a lower number of traumatic events, our research has shown a higher prevalence of traumatic events in more severe violent offenders.

The main finding of this study was that convicted male offenders with high levels of psychopathic traits were more likely to have experienced abuse and neglect during childhood, and they were even more likely to have experienced relational trauma at an early age.

Indeed, comparing our results to those reported by Nijenhuis and colleagues (2002) on the subsample of 57 male psychiatric outpatients who participated in the original validation of the TEC, the convicted offenders analysed in this study reported more childhood experiences of emotional neglect (*z*=2.7, *p*=0.008) and sexual abuse (*z*=2.2, *p*=0.027); also, a higher prevalence of physical abuse and emotional abuse was observed in our offender sample, but gender differences on these variables were not reported on the TEC validation article, thus a comparison between the two samples is not possible in this case. However, when considering other robust and well-validated measures of child neglect and abuse, such as the Childhood Experience of Care and Abuse (Bifulco, Brown, & Harris, 1994), it was observed that the sample described in this study reported a very higher prevalence of emotional neglect, emotional abuse, physical abuse, and sexual abuse (all *p*<0.001) with respect to the normal sample who participated in the Italian CECA validation study (Giannone et al. 2011).

Furthermore, almost every subject in our HRP group had experienced a trauma before the age of 10, a threshold selected for its consistency with the Italian educational system. It is to be underlined that this result does not mean that child abuse and neglect lead to psychopathy; however, it means that it is very unlikely that subjects who show severe psychopathic traits did not experience abuse and neglect in childhood. This can positively inform their assessment and treatment.

This study has some limitations to be addressed: first, the small sample size prevented the use of more complex statistical analyses, thus not allowing for a generalisation of results. Second, even though the TEC is a valid and reliable measure, the use of a self-reported measure for assessing childhood trauma may produce biased results, although there were alleged reports of adverse childhood experiences for most of the sample. Hence, there is a need for further research on the heterogeneity of psychopaths in relation to a history of trauma.

Despite these limitations, data suggest that exposure to early relational trauma can play a relevant role in the onset of violent offending behaviour, and this can be related to an early age of exposure to abuse and neglect and the subsequent development of psychopathic traits.

It is beyond the scope of this study to advance a deterministic explanation of the link between trauma and psychopathy; however, it may be assumed that traumatic memories built upon abuse, material neglect, and lack of emotional care could be responsible for a fragmented self (Meloy, 2001; Schimmenti, 2012), which is dysregulated on a psychobiological level and needs to have power and control over others through manipulation, deception and even violence.
